# Restaurant Advertising Expenditure Patterns in US Counties by Race, Ethnicity, and Income

**DOI:** 10.1007/s11524-025-01039-x

**Published:** 2026-02

**Authors:** Briana Joy K. Stephenson, Sara N. Bleich, Dania V. Francis, Keren M. Horn

**Affiliations:** Department of Biostatistics, Harvard T.H. Chan School of Public Health, Boston, MA, USA; Department of Health Policy and Management, Harvard T.H. Chan School of Public Health, Boston, MA, USA; Department of Economics, University of Massachusetts at Boston, Boston, MA, USA; Department of Economics, University of Massachusetts at Boston, Boston, MA, USA

**Keywords:** Restaurant advertising, Advertising expenditure, Targeted advertising, County-level demographics

## Abstract

Recent growth in the share of household dollars spent on food consumed outside the home, in combination with growing obesity disparities, has heightened interest in food advertising by geography and population demographics. This study aimed to identify changes in advertising expenditure by US restaurant chains across counties, grouped by income, race, and ethnicity, specifically examining whether the lowest income and highest percentage of Black and Hispanic/Latino residents are disproportionately impacted by these trends. Using expenditure and location data for the top-100 grossing US restaurant chains and county-level demographic information, we measured patterns and trends in advertising expenditure at the county level. US counties were split by population density and socio-demographics. Quantile regression analysis was performed to identify baseline differences in total spending within county groups, as well as changes over time. Results show that fast food restaurant chains reported the highest expenditures among the types of restaurants and that the majority of advertising dollars were spent on television advertisements within the highest density counties. Our results show that—for all density types—the lowest levels and the greatest declines of advertising dollars occur among high-income counties and those with a low proportion of Black and Hispanic/Latino residents. Within the lowest density (rural) counties, the highest expenditure rates occurred in counties with a high proportion of Black and Hispanic/Latino residents, regardless of income. Among the highest density (urban) counties, the highest spending levels were observed in low-income counties. Together, these results suggest that restaurant advertising dollars in high- and low-density counties are consistently targeted toward populations who are also at greater risk for obesity in the United States.

## Introduction

Since 2010, there has been a growing shift in the American food environment where dollars spent on consumed food away-from-home (FAFH) have exceeded that of food consumed at home [1]. The USDA defines FAFH as food and drink prepared outside the home for immediate consumption [2]. This gap has continued to widen, such that in 2023 over 58% of total food spent was sourced to a FAFH establishment [3]. Of FAFH establishments, restaurants accounted for the majority of dollars spent by consumers (71%, 2017–2024) [4]. The number of restaurants in the United States is increasing, with fast food restaurants outpacing all other restaurant types [5]. Given this growth in restaurant spending, combined with the high national obesity rates [6], we examine whether areas with individuals who are most at risk for obesity are disproportionately impacted by the changing trends in restaurant advertising, specifically low income and a high percentage of Black and Hispanic/Latino residents. Understanding patterns in restaurant advertising is increasingly important as it influences the American diet and overall food choices [7, 8]. For example, experimental evidence documents the effect of food advertising on children’s dietary intake, showing that children exposed to more food advertising ate more and were more likely to have obesity [9–11]. There is also research documenting differences in consumer food purchasing by race/ethnicity and socioeconomic status (SES), where communities with low income and Black or Hispanic/Latino residents were more likely to consume energy-dense, processed, and unhealthy foods. Residents in rural communities are often susceptible to limited access to healthy food options. These differences are further compounded when intersected with both low income and Black or Hispanic/Latino residents [12], as well as differences in overall food marketing [13], but much less is known about similar patterns for restaurant advertising. Additionally, restaurant food items, in comparison to food cooked and consumed at home, are often associated with larger portion sizes and a higher caloric and fat intake, which are significant risk factors for obesity [14, 15]. Overall, restaurant advertising was estimated to be about $9 billion in 2022, exceeding advertising expenditures on packaged food and beverages. This highlights that restaurants represent the single largest share of food-related advertising in the US market [16].

Different advertising mediums are often used to broaden the reach of exposure and target certain demographics. For example, outdoor ads (e.g., billboards, bus benches, subways, store fronts) are more likely to be seen by commuters and/or people reliant on public transportation. TV ads can be targeted based on the demographic likely to view certain programming. Prior research has shown that advertising of unhealthy foods, such as by fast-food restaurants, is more concentrated in communities with low income and/or a high percentage of Black or Hispanic/Latino residents and is associated with changes in body mass index (BMI) [15–18, 20]. Researchers have also documented that exposure to outdoor advertising, specifically billboards, bus benches, bus shelters, storefronts and subways differs by income, race and ethnicity [21–25], and could also vary by the population density, or urbanicity, of the geographic area being marketed. However, none of these studies focus on differential advertising patterns of restaurants beyond fast-food.

Our work builds on this knowledge and seeks to broaden our understanding of how restaurant advertising expenditure changes over time, across multiple advertising mediums, by geography, race, ethnicity, and income. With the availability of quarterly advertising expenditure data across different media and geographic locations for the top-grossing restaurant chains in the United States, our study utilizes an objective measure of local per capita restaurant advertising, building on the empirical approach of Bleich et al. [20] to examine how changes in restaurant advertising expenditure vary at the county level, accounting for differences defined by county-level income, population density, race and ethnicity. This measure has been previously used in the literature to show a modest but statistically significant association between per capita restaurant advertising expenditures and BMI for residents of low-income counties [20]. By relying on this standardized advertising expenditure measure, we are able to measure the possible exposure of these advertisements relative to population size.

## Methods

### Description of Data

Our study focused on the top 100 grossing US restaurant chains from 2012 to 2016, as this represents a significantly large share of the restaurant landscape. Restaurant chains were identified by annual rankings from the Nation’s Restaurant News [26]. Since rankings change from year to year, a total of 107 restaurant chains were included for analysis. Restaurant chains were classified into three categories: fast food (*n* = 49), fast-casual (*n* = 17), and full-service (*n* = 41). Full-service was defined as a restaurant that provides table service. Fast food and fast-casual were distinguished based on a list of criteria: (1) no table service available, (2) non-disposable utensils provided, (3) on-site food preparation, and (4) commitment to higher quality, sustainability, and fresh ingredients. Fast-casual restaurants met at least 2 of these criteria. Fast food restaurants met less than 2 of these criteria. Though we have significantly fewer restaurants in the fast-casual category, we report results for this set of restaurants as they are priced much more similarly to fast food restaurants but tend to have healthier menus and thus may have different advertising strategies. Supplementary A provides a list of all restaurant chains included and their classifications.

**Fig. 1:**
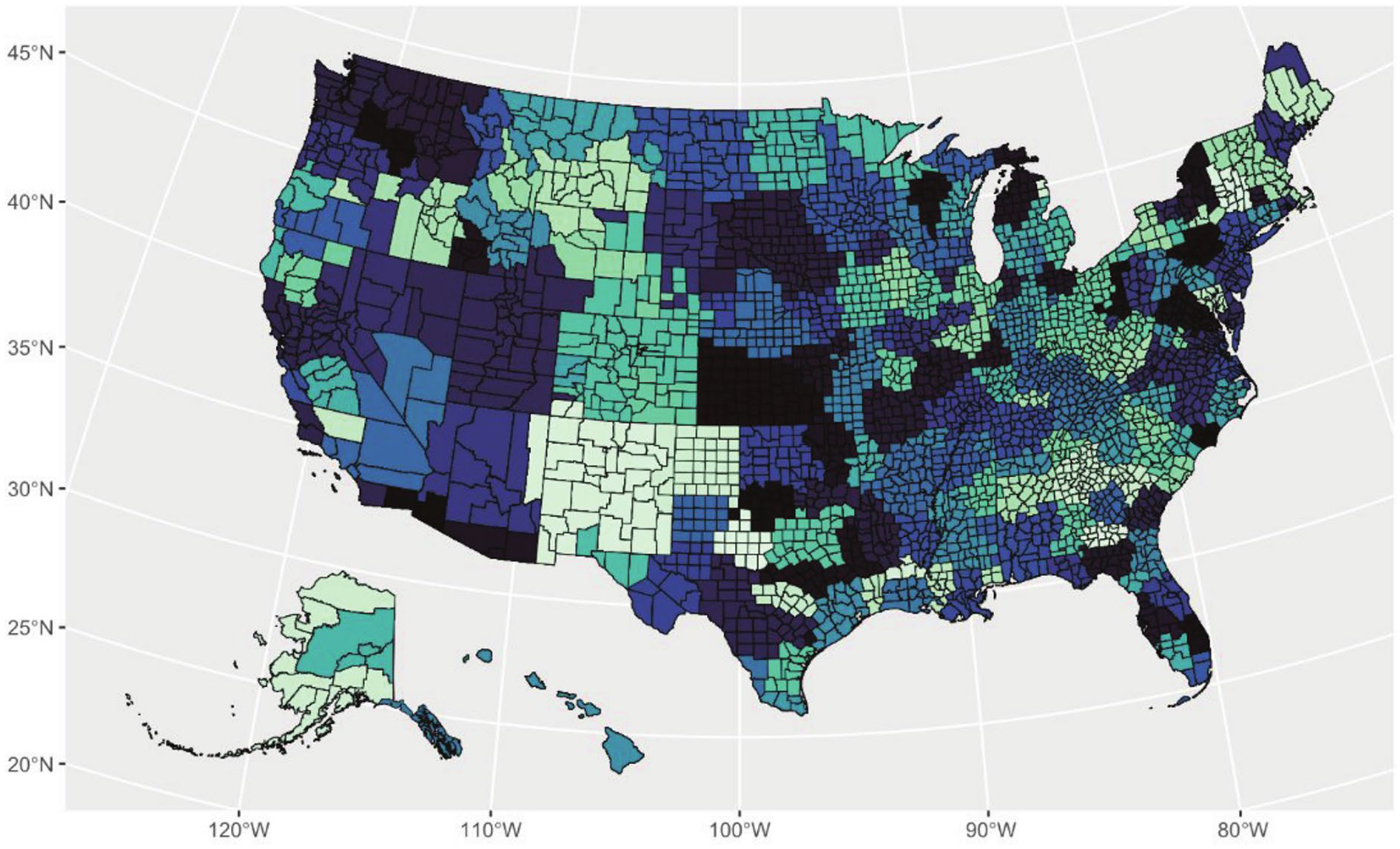
Map of 209 Designated Marketing Areal units (DMAs), which are geographically defined by Nielsen Ad Intel and separated by different colors. County boundaries are overlayed and distinguished with black border lines

The outcome of interest, per capita restaurant advertising (PCRA), was a derived measure calculated from three different data sources. (1) Geographic address locations of each restaurant chain were obtained from AggData (www.aggdata.com). (2) Data on quarterly total advertising expenditure by each restaurant chain on all media types (TV, Print, Web, Radio and Other) in each Designated Market Area (DMA) were obtained from Nielsen. DMAs are proprietary boundaries defined by Nielsen and updated annually. They include groups of counties that reflect actual patterns of TV viewing behavior. For example, a New Hampshire county may be part of the Boston DMA if the largest share of households in that county primarily watch Boston-based television stations [28]. Using DMA-level data aligns with how advertising is bought and sold, as all restaurant advertising contracts are negotiated and tracked at this level [20, 29]. Boundaries of each DMA are presented in Fig. 1, which can span multiple counties. (3) County population data were drawn from the U.S. Census Bureau’s 2012–2016 American Community Survey (ACS). Variables include the proportion of Black and Hispanic/Latino (BHL) residents by county, median income, proportion of residents with at least a 4-year college degree, unemployment rate, and county square area to calculate county population density. Further details on the three data sources used can be found in Supplementary B.

Our outcome, per capita restaurant advertising, described in the equation below, is calculated as a ratio, where the numerator consists of the ratio of the number of restaurants in a given county *c* divided by the number of restaurants in that county’s DMA, indexed by *d*, multiplied by the total DMA expenditure for that restaurant in each year, *y*, and quarter, *q*. The denominator consists of the county population for the observed year.


$${\text{PCRA}}_{cq}=\frac{\sum_{i}\frac{\text{Restaurant}\;{\text{location}}_{icy}}{\text{restaurant}\;{\text{location}}_{idy}}\times \text{total}\;\text{DMA}\;{\text{expenditure}}_{idq}}{\text{county}\;{\text{population}}_{cy}}$$

Intuitively, the numerator takes the total expenditures by a particular restaurant chain in a DMA and spreads it out across the counties in that DMA in proportion to the number of restaurants that chain has in each county. These weighted restaurant expenditures were then summed over the 107 restaurants in the sample. A county PCRA measure only includes advertising expenditures for a particular restaurant chain if that chain has a location in that county. This calculation produces a county-level measure of total restaurant advertising expenditure which is then scaled by the county population.

### Statistical Analysis

Descriptive statistics were calculated for each restaurant chain, media type, and county characteristics. Counties were classified by population density (population per square mile) and divided into quartiles. For ease of discussion, we use shorthand labels for each quartile, which describe the counties’ density. The first quartile consisted of counties with the lowest density (rural). The second quartile contained low-density (suburban-1) counties. The third quartile contained moderate density (suburban-2) counties. The fourth quartile contained the highest density (urban) counties. All 3,141 US counties were included for analysis.

Counties were further classified demographically, to highlight the cross-section of income and race/ethnicity [12]. Four categories were defined based on ACS-obtained information of resident composition and median income level at baseline (2012). County groups were first stratified based on which counties had proportions of Black and Hispanic/Latino (BHL) residents above or below the overall median (10%), labeled as *High-BHL* and *Low-BHL* respectively. County groups were further stratified based on which counties had a median income above or below the 2012 US median ($47,220), labeled as *High-Income* and *Low-Income*, respectively.

To identify changes in PCRA expenditure over time, a linear quantile mixed model was run, with PCRA expenditure treated as the primary outcome and county type (Income/BHL) as the primary exposure. Implementation of a quantile mixed model allowed us to account for outlying restaurant chains that observed considerably higher or lower PCRA expenditure dollars. County-level covariates were included in the model to adjust for the proportion of residents with at least a bachelor’s degree and the unemployment rate. Additional fixed effects were included to adjust for time (indexed as quarterly-year) and election year to account for potential changes in advertising trends driven by political cycles (2012, 2014, 2016). An interaction term of Income-BHL county type and time was also included to account for temporal variation by county type. Due to the repeated measures observed for each county, within-county variation was adjusted for via a random intercept indexed at the county level. To account for differences in patterns and trends by population density, regression analysis was stratified according to the four population density quartiles, resulting in four sets of estimates. A subsequent stratified analysis was performed to examine if expenditure differences and changes over time were observed by restaurant type (fast food, fast-casual, full-service).

Data analysis was processed in STATA, R and Python software. Statistical analysis and visualizations were performed in R using the following packages: dplyr [30], nlme [31], lqmm [32], ggmap [33], and ggplot2 [34].

## Results

Table 1 describes the median per capita restaurant advertising expenditure for the 107 restaurant chains. Overall, PCRA had a slight decline from 2012 to 2015, from $3.59 to $3.43, but reversed its trend in 2016 ($3.56). For an average-sized county in the United States, with approximately 100,000 residents, this is equivalent to $350,000. For more populous urban counties, with at least 1 million residents, this is equivalent to at least $3.5 million. By restaurant type, fast-food restaurants had the highest PCRA expenditure with an average of $2.81 over the 5-year timespan, while fast-casual restaurants had the lowest. TV advertising had the highest median PCRA expenditure across media types; thus, our results can largely be seen as capturing differences in TV advertising trends. Radio advertising had the lowest median PCRA expenditure.

Table 2 describes baseline county characteristics across the four population density quartiles. In 2012, the counties with the highest density were also the counties with the highest median income and the highest proportion of residents with 4 years of college or more. The mean proportion of Black and Hispanic/Latino residents ranges from a low of 15.4% in the moderate density (suburban-2) counties to a high of 21.9% in the highest density (urban) counties. This indicates a positive skew in our distribution of Black and Hispanic/Latino residents, such that there are counties with much higher proportions of this residential demographic pulling the means above the overall median of about 10%.

Within the population density groupings, we observed significant variation in the income/BHL makeup of the counties. Over half of the high-income/high-BHL counties were in the highest density (urban) counties. By contrast, low-income/high-BHL counties were more evenly distributed across all density groups.

**Fig. 2:**
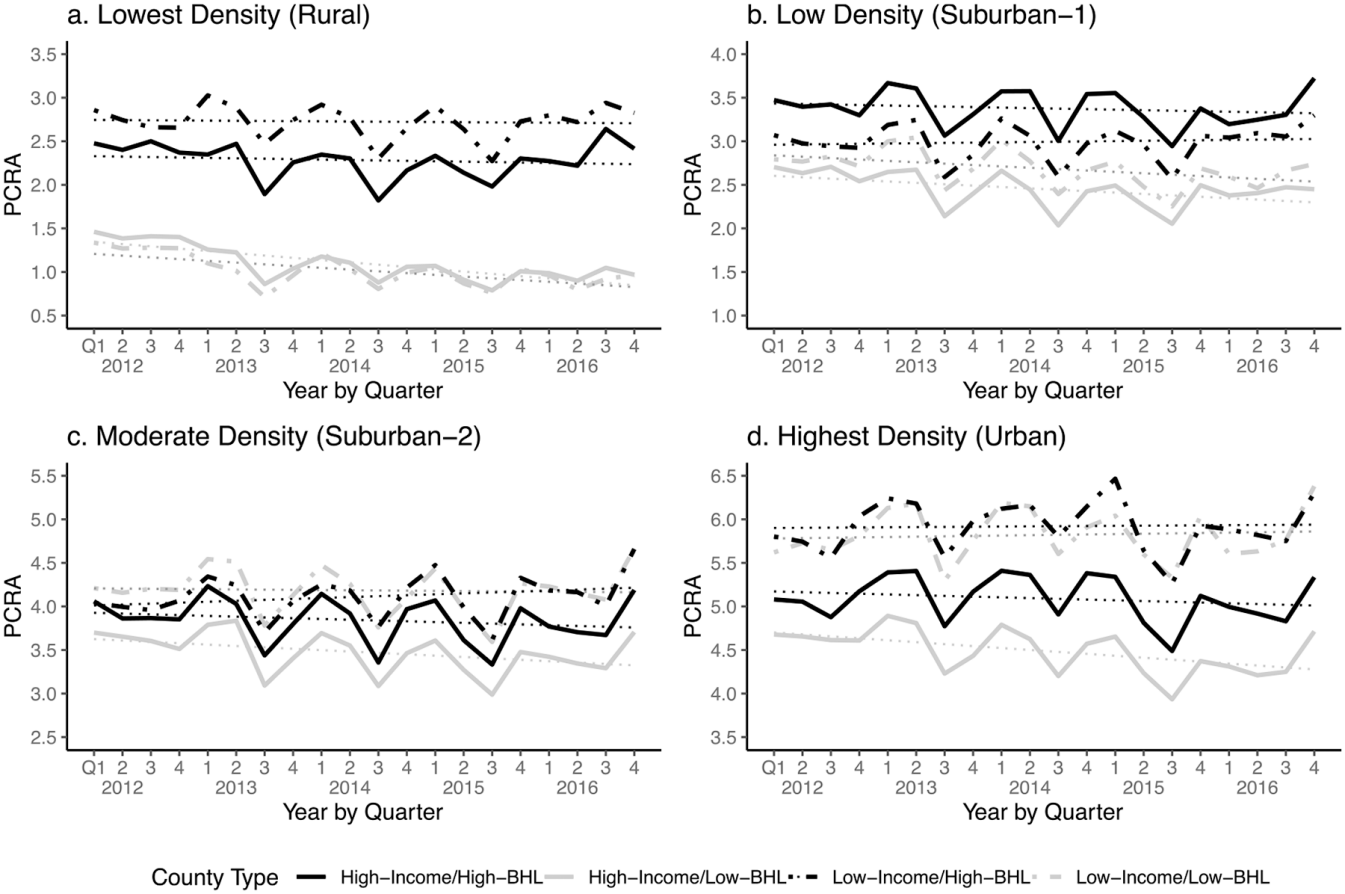
Patterns and trends over time of median per capita restaurant advertising in dollars, stratified by county population density. Note: Solid lines denote counties with median income above the national average. Dashed lines denote counties with median income below the national average. Y-axis ranges differ across panels to allow clear visualization of trends within each advertising category

The results of our primary analysis are presented in Fig. 2 and Table 3. Figure 2 provides a visualization of how the median per capita restaurant advertising changed over time by county (Income/BHL) type across the four population density quartile groups, unadjusted for confounders. Results for each density group are displayed in separate panels. Given the differences in the absolute levels of advertising across these geographies, the Y-axis ranges (PCRA) differ for each panel to prevent compression of the variation. Additionally, there appears to be some seasonality in these data, with a clear and consistent drop in the third quarter. These trends are documented in the literature and may represent the quieter nature of summer as well as the holiday ramp-up that occurs in the fourth quarter [35, 36]. Table 3 provides the parameter estimates from the linear quantile mixed model, which are adjusted for confounders. Results from these two analyses confirm the same set of findings.

Amongst the lowest population density (rural) counties, High-BHL counties, regardless of income, had higher restaurant advertising expenditures than Low-BHL counties. Over time, the expenditures in High-BHL counties appeared stably high, while those in Low-BHL counties appeared to decrease slightly (Fig. 2a). These trends are supported statistically, after adjusting for key confounders (Table 3, column 1). Compared to the reference group, Low-BHL county subgroups indicated an increase of $1.50, but changes over time were not statistically significant.

For low population density (suburban-1) counties, differences were still large between high-income counties along lines of BHL, but there were no longer differences between low-income counties along lines of BHL. High-income/high-BHL counties exhibited the highest levels of advertising expenditures, and high-income/low-BHL counties exhibited the lowest levels of expenditures (Fig. 2b). After adjusting for confounders, high-income/high-BHL counties had about $1.46 more median advertising expenditures than high-income/low-BHL counties (Table 3, column 2). Unlike the rural counties, there was no statistically significant differentiation in expenditures among low-income counties by high- or low-BHL status. Trend-wise, spending in these low-density suburban counties decreased slightly but significantly, except for low-income/high-BHL counties where advertising expenditures were flat.

For moderate population density (suburban-2) counties, no statistically significant differences in the median levels of expenditures by income or Black and Hispanic/Latino status were observed. Over time there was a slight increase in expenditures in low-income counties relative to high-income counties, regardless of Black and Hispanic/Latino status. For example, the coefficients on the low-income county indicators interacted with time indicated an increase of one to three cents per quarter in low-income counties relative to the reference group of high-income/low-BHL counties which saw a decrease in spending of about one cent per quarter over the sample time frame (Table 3, column 3).

For the highest population density (urban) counties, differences among high- and low- income counties were more pronounced, where lower-income counties experienced higher advertising spending compared to higher-income counties regardless of Black and Hispanic/Latino status (Fig. 2d). After adjusting for confounders, advertisers spent about $1.32 more per capita in low-income/low-BHL urban counties than in high-income/low-BHL urban counties and about $1.12 more in low-income/high-BHL urban counties (Table 3, column 4). Differences between high- and low-BHL counties with high incomes were not statistically significant. Adjusted trends in urban counties showed high-BHL counties, rather than the low-income counties, had statistically significant differences. While advertising expenditures in high-income/low-BHL counties decreased by about two cents per quarter, expenditures in high-BHL counties showed a minimal decrease in comparison (Table 3, column 4). Thus, among urban counties, the differences in levels of spending were largest by income status while the differences in trends of spending over time were largest by Black and Hispanic/Latino status. To summarize, the largest gaps in patterns and trends across county types emerge in rural and urban counties, where the dividing line is race and ethnicity in rural counties, and the dividing line is income in urban counties.

Table 4 provides the regression output for each restaurant type across the different population density groups. Overall, results at the fast-food level were consistent with those seen in the pooled analysis. In rural counties, significance was only found among fast-food restaurants, consistent with results in Table 3. Statistical significance was not evident for fast-casual and full-service restaurants.

Some differences emerged from the overall analysis in low population density (suburban-1) counties. Fast-food restaurant levels of advertising spending were significantly different across all three comparative income/BHL groups, whereas in pooled results differences only emerged between high-income/high-BHL and high-income/low-BHL. Temporal effects within low-income/high-BHL counties for fast food restaurants were consistent with overall results. In contrast, in suburban-1 counties fast-casual restaurants increased spending relatively more in high-income/high-BHL counties and low-income/high-BHL counties, relative to high-income/low-BHL counties, but magnitudes here were small. No significant differences were found among full-service restaurants.

Moderate population density (suburban-2) counties had consistent results with the overall analysis among fast-food restaurants, with additional significance noted in the temporal effect of high-income/high-BHL counties. Changes in spending within fast-casual restaurants were only significantly different from the baseline group within low-income/high-BHL counties. Levels of spending within full-service restaurants were only significantly different from the baseline group within high-income/high-BHL counties, consistent with the overall analysis, with no significant differences in changes over time.

The highest population density (urban) counties had the most different results from the overall analysis. Differences in levels of spending among fast-casual and full-service restaurants were mostly consistent with overall results, but we no longer observed differences in levels of spending across county types within fast food restaurants. In terms of changes over time, we found similar results across all restaurant types, with the largest differences emerging within fast food restaurants.

## Discussion

This study identified county-level changes in advertising expenditure by US restaurant chains by income, race and ethnicity. While no significant changes over time were noted in our results, patterns did emerge by restaurant type, population density and county-level sociodemographic characteristics. Fast food restaurant chains reported the highest expenditure, and most advertising dollars were spent on television advertisements. Stratified by population density, expenditure patterns varied by income and the concentration of Black or Hispanic/Latino residents. Specifically, rural counties had higher restaurant expenditure rates in counties with a high level of Black and Hispanic/Latino residents. Urban counties had higher restaurant expenditure rates in low-income counties. An intersection of demographics was most apparent in suburban counties where higher restaurant expenditure was observed among counties with low income and a high level of Black and Hispanic/Latino residents whereas lower restaurant expenditures were observed among counties with high income and a low level of Black and Hispanic/Latino residents. These results suggest that restaurant advertising dollars in high-density counties are consistently targeted toward county populations that are at greater risk for obesity.

Within fast-casual restaurants, the largest difference in restaurant expenditure was observed in urban counties characterized by high income with a high level of Black and Hispanic/Latino residents compared to those characterized by high income with a low level of Black and Hispanic/Latino residents. Within full-service restaurants, the largest differences in restaurant expenditure were observed by county income. Further research is needed to understand what factors are driving these differences for fast-casual and full-service restaurants within certain types of counties.

Few studies have examined differences in restaurant advertising by medium type (e.g., TV or print) and by county characteristics [13]. To our knowledge, no study to date has focused specifically on advertising expenditure across the restaurant landscape of the highest grossing chains including all possible media and restaurant types. Further, while most studies relied on a cross-sectional design, our data examined longitudinal trends for the entire country. This was achieved by the development of our objective per capita restaurant advertising measure, adapted from Bleich et al. [19]. Our results were consistent with prior research but provide greater context with the inclusion of different restaurant types.

Despite these novel findings, our study contained some limitations. First, our analysis focused on the top 100-grossing US restaurant chains. While this does cover a significantly large share of the restaurant landscape, it does not provide a comprehensive picture of the restaurant landscape. Independent restaurants and regional chains were not included and may differ in advertising strategies from the larger chains included in this analysis. Circana reports that the top 50 restaurant chains account for approximately 61% of all US restaurant spending. Therefore, we are confident that our analysis provided an accurate reflection of the overall national restaurant advertising landscape across the US [27]. Second, our analysis was constrained by data availability. Working across multiple national databases constrained our study to 2012–2016 data, which were available across all sources and effectively harmonized. Still, the results offer a comprehensive picture of a complex relationship between restaurant expenditures, geography and population characteristics that is not currently available in the published literature. Today, overall spending on food outside the home is more than 30 percent greater than food at home. Of the top 100 restaurant grossing chains analyzed in our study, 81 chains are still among the top-100 grossing, illustrating the relevance of our work still today [37].

Another limitation is that our analysis relied on advertising expenditures, which do not necessarily translate to exposure, or how much people directly interact with advertisements. Different media types vary in cost, which may not directly correlate with exposure. For example, web/online advertising is lower in cost but has a larger potential for audience reach compared to TV advertising which has the highest expenditure of all media types [38]. Despite these differences in cost by media type, during the study period, TV was the primary mode used by restaurant chains and these advertisements were disproportionately targeted toward certain communities. The last decade has seen an exponential increase in mobile device use as well as social media use. This has generated a major shift by advertisers toward digital platforms [38]. After 2016, web/digital advertising expenditure trends exceeded and outpaced those of TV advertising [41, 42]. As the first study to examine advertising expenditures of top-grossing restaurant chains in the United States, our study period provides a critical baseline of how restaurants targeted TV advertising compared to other media types in specific communities along race, ethnicity, and income. TV exposure is measurable through Nielsen’s Gross Rating Points (GRP) system. However, research measuring exposure to digital/web and other media types has varied [39, 40]. To our knowledge a validated metric to measure digital/web exposure has not yet been established. Further research is needed to generate a standardized measure of exposure to determine if exposure patterns are consistent with expenditure patterns.

Even with a more robust measure of exposure, this approach still only measures one dimension of a restaurant’s marketing strategy. Restaurants which utilize other advertising mediums, such as print ads in strategic commuter-friendly locations or partnership with digital technologies may be targeting certain populations. More work is needed to understand integrated marketing approaches of restaurant chains and how they have shifted toward the more dominant digital advertising revenue stream over the last 10 years. Lastly, while exposure to different ads may differ by age, we did not consider age in our analysis. The relationship of age and obesity is individualized and complex. To avoid ecological fallacies being made, we did not include it in our county-level analysis. Future research that can obtain individual demographic information is positioned to address age’s mediating role within the food-obesity relationship.

This is the most rigorous study to date looking at patterns in advertising expenditures among the top 100 grossing restaurants by geography and population demographics. Our results suggest that restaurant advertising dollars in high- and low-density counties are consistently targeted toward populations at greater risk for obesity in the United States. While efforts have been made to encourage restaurants to improve food offerings on their menu, little change has yet been found in the overall nutritional value of these restaurants [43–47]. Policies that implemented a sugary beverage tax have decreased the volume of sales on those products [48]. Similar policies may show promise at a broader scale for restaurants with unhealthy food options. This further emphasizes the relevance of our work and the need for effective policies to address the widening obesity disparities in targeted communities. It also underscores important future research such as evaluating the impact of chain restaurant advertising on major chronic disease risk factors, such as obesity, across US counties.

## Supplementary Material

Supplementary Materials

**Supplementary Information** The online version contains supplementary material available at https://doi.org/10.1007/s11524-025-01039-x.

## Figures and Tables

**Table 1:** Median per capita restaurant advertising expenditures by restaurant and media type, in dollars

	2012	2013	2014	2015	2016
*Overall*	$3.59	$3.56	$3.55	$3.43	$3.56
*Restaurant type*					
Fast food	$2.80	$2.78	$2.85	$2.78	$2.85
Fast-casual	$0.00	$0.00	$0.00	$0.00	$0.00
Full-service	$0.55	$0.52	$0.45	$0.39	$0.47
*Media type*					
TV	$3.32	$3.28	$3.30	$3.17	$3.32
Print	$0.04	$0.05	$0.04	$0.02	$0.01
Web	$0.02	$0.02	$0.02	$0.03	$0.04
Radio	$0.06	$0.03	$0.02	$0.01	$0.00
Other	$0.05	$0.05	$0.05	$0.06	$0.05

The sample includes 3141 US counties

**Table 2:** Baseline county characteristics stratified by population density

	Overall	Lowest density (Rural)	Low density (Suburban-1)	Moderate density (Suburban-2)	Highest density (Urban)
*Mean estimate*					
Population density, ppl/mi2	364.1	6.7	29.3	71.1	1348.3
Median income, in $000’s	$47.22	$45.77	$42.38	$45.02	$55.69
Black or Hispanic/Latino, %	17.9%	16.2%	18.3%	15.4%	21.9%
Unemployment, %	6.4%	5.6%	7.0%	6.8%	6.2%
4 years college or more, %	20.7%	19.1%	16.5%	18.4%	29.0%
*County sample type (count, N)*					
Low-income/high-BHL	815	189	266	216	144
Low-income/low-BHL	757	227	241	224	65
High-income/high-BHL	717	102	88	125	402
High-income/low-BHL	852	267	190	220	175
*N*	3141	785	785	785	786

Mean estimates reflect county characteristics in 2012 by neighborhood density. Sample count represents the number of counties in our sample that are identified in each density group

**Table 3:** Quantile regression estimates reflecting the association between county income/BHL category and restaurant advertising expenditures

	Lowest density (Rural)	Low density (Suburban-1)	Moderate density (Suburban-2)	Highest density (Urban)
Low-income/high-BHL	1.50 (0.35)***	0.66 (0.36)	− 0.07 (0.43)	1.12 (0.29)***
Low-income/low-BHL	− 1.84 (0.21)	0.48 (0.23)	0.44 (0.43)	1.32 (0.53)*
High-income/high-BHL	1.50 (0.41)**	1.46 (0.46)*	0.50 (0.37)	0.43 (0.33)
Time	0.00 (0.00)	− 0.02 (0.01)**	− 0.01 (0.01)*	− 0.03 (0.01)**
Low-income/high-BHL*time	0.00 (0.00)	0.01 (0.01)**	0.03 (0.00)***	0.02 (0.01)**
Low-income/low-BHL*time	0.00 (0.00)	0.00 (0.00)	0.01 (0.00)***	0.01 (0.01)
High-income/high-BHL*time	0.00 (0.00)	0.01 (0.01)	0.01 (0.01)	0.01 (0.00)**
Intercept	1.42 (0.28)***	2.81 (0.41)***	4.23 (0.45)***	5.58 (0.59)***

Confidence intervals are in parentheses. Advertising is measured using PCRA. Time is defined as year/quarter. Adjusted for county-level proportion of residents with more than 4 years of college education, county-level unemployment rate, election year indicator, and quarter-fixed effects

*BHL* Black and Hispanic/Latino

*** *p* < 0.01;

** *p* < 0.05;

* *p* < 0.1

**Table 4:** Quantile regression estimates reflecting the association between county income/BHL category and restaurant advertising expenditures, by restaurant type

	Lowest density (Rural)	Low density (Suburban-1)	Moderate density (Suburban-2)	Highest density (Urban)
*Fast food*				
Low-inc/high-BHL	1.36 (0.21)***	1.16 (0.31)***	0.56 (0.30)	0.08 (0.28)
Low-inc/low-BHL	0.00 (0.03)	1.01 (0.32)**	0.62 (0.39)	0.08 (0.38)
High-inc/high-BHL	1.36 (0.21)***	1.13 (0.35)**	0.24 (0.32)	0.07 (0.25)
Time	0.00 (0.00)	− 0.01 (0.01)*	− 0.01 (0.01)	− 0.01 (0.01)
Low-inc/high-BHL*time	0.00 (0.00)	0.01 (0.01)*	0.02 (0.00)***	0.03 (0.00)***
Low-inc/low-BHL*time	− 0.00 (0.00)	0.00 (0.00)	0.01 (0.00)**	0.02 (0.01)**
High-inc/high-BHL*time	− 3.50 (0.00)***	0.00 (0.01)	0.01 (0.00)**	0.02 (0.00)***
Intercept	1.30 (0.11)***	1.85 (0.44)***	3.46 (0.39)***	4.75 (0.43)***
Fast-casuals				
Low-inc/high-BHL	− 0.00 (0.01)	− 0.01 (0.01)	0.02 (0.01)	− 0.01 (0.02)***
Low-inc/low-BHL	− 0.00 (0.01)	− 0.01 (0.01)	0.00 (0.01)	0.07 (0.01)
High-inc/high-BHL	0.00 (0.01)	0.04 (0.01)**	0.02 (0.01)	0.66 (0.12)***
Time	0.00 (0.00)	0.00 (0.00)***	0.00 (0.00)**	0.00 (0.00)***
Low-inc/high-BHL*time	0.00 (0.00)	0.00 (0.00)**	0.00 (0.00)***	0.00 (0.00)***
Low-inc/low-BHL*time	0.00 (0.00)	0.00 (0.00)	0.00 (0.00)	0.00 (0.00)**
High-inc/high-BHL*time	0.00 (0.00)	0.00 (0.00)*	0.00 (0.00)	0.00 (0.00)***
Intercept	− 0.03 (0.01)*	− 0.08 (0.02)***	− 0.09 (0.03)**	0.05 (0.04)
Full-service				
Low-inc/high-BHL	0.25 (0.16)	0.10 (0.17)	0.08 (0.23)	0.74 (0.17)***
Low-inc/low-BHL	0.07 (0.12)	0.39 (0.20)	0.19 (0.15)	0.71 (0.35)*
High-inc/high-BHL	− 0.00 (0.18)	0.18 (0.23)	0.39 (0.19)*	− 0.00 (0.00)
Time	0.00 (0.01)	0.00 (0.01)	− 0.01 (0.00)**	− 0.03 (0.00)***
Low-inc/high-BHL*time	− 0.02 (0.01)	− 0.00 (0.01)	0.00 (0.00)	− 0.01 (0.00)*
Low-inc/low-BHL*time	− 0.00 (0.01)	− 0.00 (0.01)	0.00 (0.00)	0.00 (0.00)
High-inc/high-BHL*time	0.01 (0.01)	− 0.01 (0.01)	− 0.00 (0.00)	− 0.00 (0.00)
Intercept	0.89 (0.34)*	0.29 (0.19)	0.22 (0.24)	1.84 (0.30)***

Standard errors are in parentheses. Advertising is measured using PCRA. Time is defined as year/quarter. Adjusted for county-level proportion of residents with more than 4 years of college education, county-level unemployment rate, election year indicator, and quarter-fixed effects

*BHL* Black and Hispanic/Latino

*** *p* < 0.01;

** *p* < 0.05;

* *p* < 0.1

## Data Availability

Location data of restaurant chains is available through AggData (www.aggdata.com) for purchase. Advertising expenditure data was obtained from Nielsen Ad Intel via a partnership with the University of Chicago Booth School of Business. This data is not publicly available but may be made available through a subscription contract with the Kilts Data Center for Marketing.
