# Supplementary material for: Restaurant Advertising Expenditure Patterns in US Counties by Race, Ethnicity, and Income
Source: J Urban Health. Author manuscript; Available in PMC 2026 May 10. (PMC13136477; doi:10.1007/s11524-025-01039-x)
Supplement: Supplementary Materials [file NIHMS2171893-supplement-Supplementary_Materials.docx]

# Supplementary A

**Supplementary Table 1 Top-grossing restaurant chains (by restaurant type) from 2012-2016**

| Chain Type | | |
| --- | --- | --- |
| Fast-Food (N=49) | Fast Casual (N=17) | Full Serve (N=41) |
| 7-Eleven | Boston Market | Applebee's |
| Arby's | Chipotle | BJ's Restaurant & Brewhouse |
| Auntie Anne's | Chuck E Cheese | Bob Evan's |
| Baskin Robbins | Cici's Pizza | Bonefish Grill |
| Bojangles | Corner Bakery | Buffalo Wild Wings |
| Burger King | Dickey's Barbecue Pit | California Pizza Kitchen |
| Captain D's Seafood | Jason's Deli | Capital Grille |
| Carl's Jr/Hardee's | Marco's Pizza | Carrabba's Italian Grill |
| Casey's General Store | Moe's Southwest Grill | Cheddar's |
| Checker's/Rally's | Noodles & Company | Cheesecake Factory |
| Chick-Fil-A | Panda Express | Chili's |
| Church's Chicken | Panera Bread | Cracker Barrel |
| Culver's | Papa Murphy's | Dave & Buster's |
| Dairy Queen | Potbelly | Denny's |
| Del Taco | Qdoba | Famous Dave's |
| Domino's | Round Table Pizza | Friendly's |
| Dunkin Donuts | Zaxby's | Frisch's/Bob's Big Boy |
| Einstein Bros |  | Golden Corral |
| El Pollo Loco |  | Hooters |
| Firehouse subs |  | IHOP |
| Five Guys |  | Joe's Crab Shack |
| In-N-Out Burger |  | Logan's Roadhouse |
| Jack in the Box |  | Longhorn Steakhouse |
| Jamba Juice |  | Maggiano's Little Italy |
| Jersey Mike's Subs |  | O'Charley's |
| Jimmy John's |  | Olive Garden |
| KFC |  | On the Border Mexican Cantina |
| Krispy Kreme |  | Outback Steakhouse |
| Krystal Restaurant |  | Perkins |
| Little Caesar's |  | PF Chang's |
| Long John Silver's |  | Pizza Hut |
| McAlister's Deli |  | Red Lobster |
| McDonald's |  | Red Robin |
| Papa John's |  | Romano's Macaroni Grill |
| Pollo Tropical |  | Ruby Tuesday |
| Popeye's |  | Ruth's Chris Steakhouse |
| Quizno's |  | Texas Roadhouse |
| Raising Cane's Chicken |  | TGI Friday's |
| Sbarro |  | Waffle House |
| Sheetz |  | Wingstop |
| Sonic |  | Yard House |
| Starbucks |  |  |
| Steak N Shake |  |  |
| Subway |  |  |
| Taco Bell |  |  |
| Tim Horton's |  |  |
| Wendy's |  |  |
| Whataburger |  |  |
| White Castle |  |  |

# Supplementary B: Description of Data Sources

*AggData (restaurant locations):*

Aggdata ([www.aggdata.com](http://www.aggdata.com/)) is a data service provider that provides the number and location of all chain restaurants in each US county. The data collected by Aggdata is updated every three months. Data was obtained from 2012 to 2016 for each of the 107 restaurant chains included in our analysis.

*Nielsen Ad Intel (quarterly advertising spending):*

Nielsen Ad Intel datasets include data on advertising expenditure at the Digital Marketing Area (DMA) level. This data is made available to our research team through a partnership with University of Chicago Booth School of Business, Kilts Center for Marketing. DMAs, developed by Nielsen Media Research, divide the U.S. into distinct groups of counties, which generally surround urban areas and receive the same advertising mediums. Restaurant advertising data for each chain includes cost, product description, the DMA where the advertisement was purchased, and media type (e.g., print, television, internet, radio). Data is released annually to researchers. Spending data was organized at the quarterly level consistent with the restaurant location data obtained from AggData. National advertising spending was evenly distributed across the 209 DMAs and added to the local spending reported at the DMA level.

*American Community Survey (county-level characteristics):*

County-level information about population characteristics (e.g., race/ethnicity) was obtained from the American Community Survey. The ACS is a continuous survey administered by the Census Bureau to a sample of approximately 2 million people each year. County-level information about the economic environment were obtained from the Census Small Area Income and Poverty Estimates and Bureau of Labor Statistics Local Area Unemployment data series.^50^
